# Locally Acquired Dengue Virus Infection in a Non-endemic Area

**DOI:** 10.7759/cureus.93318

**Published:** 2025-09-26

**Authors:** Tam-Dan Dinh, Michael D Yashar

**Affiliations:** 1 Medicine, University of California Los Angeles, David Geffen School of Medicine, Los Angeles, USA

**Keywords:** acute undifferentiated febrile illness, arbovirus infection, autochthonous infections, clinical case report, dengue fever, dengue virus (denv), global health, infectious disease medicine, infectious diseases epidemiology, non-endemic region

## Abstract

Dengue virus (DENV) infection occurs through the transmission of the virus via mosquito vectors that are largely endemic to subtropical or tropical regions in the world. Patients from non-endemic areas typically report recent travel history and sudden onset of high fever, accompanied by severe headache, retro-orbital pain, and nausea. We present a case of a patient with a locally acquired dengue fever infection in a non-endemic area in Southern California. An extensive medical evaluation was performed by multiple physicians in various healthcare settings, ultimately leading to this diagnosis. Though our patient's presenting constellation of symptoms was consistent with dengue fever, his lack of exposure to an area known to be endemic for DENV made this a surprising and extraordinarily challenging diagnosis. This case report demonstrates the importance of maintaining a high index of clinical suspicion for acute DENV infection in those presenting with an acute febrile illness and findings concordant with this diagnosis.

## Introduction

Dengue virus (DENV) infection is an acute mosquito-borne viral illness that is most commonly found in Southeast Asia, Latin America, Africa, and other tropical and subtropical regions worldwide [1]. The illness is spread through the bite of two mosquito species, *Aedes aegypti* and *Aedes albopictus*, which are the main vectors for transmitting DENV infections to humans and thrive in warm, humid environments [2]. *Aedes *mosquitoes utilize water-holding containers to lay their eggs, preferentially inhabiting and surrounding human dwellings, thereby increasing the likelihood of infection in highly populated areas [3]. A notable risk factor for the disease includes living in higher-temperature climates, which can facilitate viral amplification, along with virus transmission from the vector-carrying mosquito, which is linked to changes in reproduction and biting rates. Considering this, the United States Centers for Disease Control and Prevention (CDC) recommends that the public utilize Environmental Protection Agency-approved mosquito repellents during travel to and from areas with dengue transmission [4].

The pathogenesis of DENV infection is based on the interplay between the virus itself, the host genes, and the immune response. These include non-structural protein 1 (NS1) and viral antigens, along with anti-DENV NS1 antibodies and autoimmunity, which, when synergistically affected, can lead to severe DENV infection [5].

Following an incubation period of four to seven days, symptomatic patients may present with an influenza-like illness, characterized by a spectrum of symptoms, including high fever, headaches, sore throat, anorexia, and muscle and joint pain [6]. Infections can also be asymptomatic or progress to a severe form known as dengue hemorrhagic fever or dengue shock syndrome [5]. These can be characterized by hypovolemic shock, coagulation abnormalities, and capillary permeability that may lead to complications such as multi-organ failure and death. Individuals who are older, pregnant, or have diabetes mellitus have an increased risk of hospitalization following DENV infection [7]. Diagnosis and management of DENV infection can be difficult due to its heterogeneous presentation and large spectrum of associated symptoms that resemble those of many acute febrile illnesses, which can hinder early identification and consideration of effective medical treatments for accompanying symptoms.

## Case presentation

A 58-year-old male Southern California resident with a history of non-insulin-dependent diabetes, elevated transaminases, vertigo, renal colic, hypertension, hyperlipidemia, and a remote prior history of severe COVID-19 disease, where he had required intubation and hospitalization for over 30 days, followed by recovery, presented to a local emergency department (ED) for evaluation of a high fever. His symptoms began the night prior with a gradual onset of headache, generalized body aches, nausea, chills, and a fever of 39.4°C (103°F) at home. He noted intermittent right-sided flank pain and imbalance when walking. He endorsed a 7/10 severity headache pain and 4/10 severity right lower back pain. He was given 600 mg of ibuprofen and 1000 mg of acetaminophen, and he noted some relief. At the time, he denied any shortness of breath, cough, rhinorrhea, sore throat, nasal congestion, neck stiffness, photophobia, rash, abdominal pain, or emesis. He also reported no urinary symptoms, dysuria, or hematuria. He also reported some pruritus on the eyelids and discomfort in the left ear. He denied any recent travel or contact with the sick. The patient reported often gardening in his yard, where he regularly encountered mosquitoes. He also reported having a pet dog that had been outside quite frequently, though it was not known to have any obvious flea infestation.

The patient's vital signs on initial presentation were notable for a temperature of 37.4°C (99.3°F) and mild tachycardia at 105 beats per minute. His physical examination was otherwise unremarkable. A complete blood count and basic metabolic panel were performed, each of which was unremarkable except for a mildly elevated serum creatinine (Table 1).

**Table 1:** Relevant serum laboratory test values during initial emergency department presentation

Laboratory Test	Result	Reference Range
White blood cell (WBC) count	4.47 × 10^3^/µL	4.23–9.07 × 10^3^/µL
Hemoglobin	14.9 g/dL	13.7–17.5 g/dL
Platelet count	178 × 10^3^/µL	163–337 × 10^3^/µL
Serum creatinine	1.37 mg/dL	0.70–1.30 mg/dL

Other testing included a respiratory viral panel, which was negative for COVID-19, influenza, and other viral respiratory pathogens. His chest X-ray showed no acute disease, infiltrate, or effusion. He was discharged in stable condition with a diagnosis of febrile illness, presumed to be due to a viral syndrome, and without clinical concern for sepsis. He was otherwise encouraged to continue supportive measures, including oral hydration and antipyretics, and to follow up with primary care within 48 hours.

The patient subsequently presented to an urgent care clinic two days later, with ongoing fevers, a 7/10 severity headache without significant improvement, as well as continued body aches and lower right back pain. A comprehensive physical exam at this time was unremarkable, including a normal neurologic exam and no meningeal signs, aside from mild dry mucous membranes, conjunctival injection, and a temperature of 38.3°C (100.8°F). Labs at this time demonstrated a normal procalcitonin, as well as mildly elevated C-reactive protein (CRP), alanine transaminase (ALT), aspartate transaminase (AST), and lipase values. There was a mild leukopenia of 3.01, with all absolute differential cell counts within normal limits, except for a low absolute lymphocyte count. The platelet count had also reduced from 178, two days prior, to 130 (Table 2).

**Table 2:** Relevant serum laboratory test values during follow-up urgent care presentation

Laboratory Test	Result	Reference Range
White blood cell (WBC) count	3.01 × 10^3^/µL	4.16–9.95 × 10^3^/µL
Absolute lymphocyte count	0.66 × 10^3^/µL	1.30–3.40 × 10^3^/µL
Hemoglobin	16.2 g/dL	13.5–17.1 g/dL
Platelet count	130 × 10^3^/µL	143–398 × 10^3^/µL
Serum creatinine	1.23 mg/dL	0.60–1.30 mg/dL
Alanine transaminase (ALT)	157 U/L	8–70 U/L
Aspartate transaminase (AST)	246 U/L	13–62 U/L
Serum lipase	159 U/L	13–69 U/L
Erythrocyte sedimentation rate (ESR)	4 mm/hr	<12 mm/hr
C-reactive protein (CRP)	2.2 mg/dL	<0.8 mg/dL
Procalcitonin	0.24 µg/L	0.1–0.5 µg/L

The patient's infectious mononucleosis antibody and HIV antigen/antibody testing were also negative. Despite receiving intravenous fluids and acetaminophen in the clinic, the patient continued to feel unwell, and so he was advised to immediately return to the ED for further evaluation and treatment.

**Figure 1:**
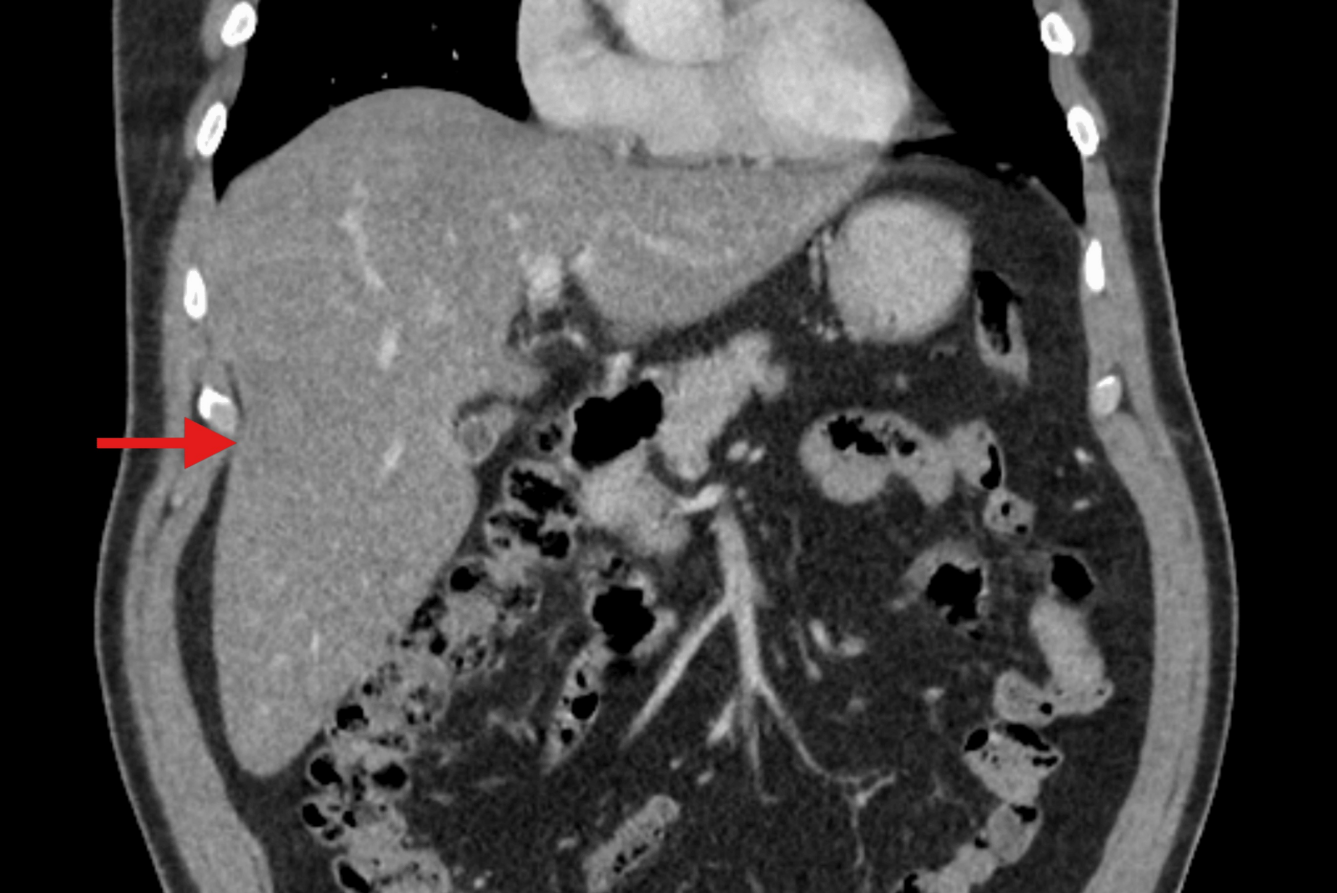
CT abdomen and pelvis with contrast Hepatomegaly with elongated right hepatic lobe measuring 23 cm in the craniocaudal axis (red arrow).

Further testing performed in the ED included unremarkable hepatitis antibody and antigen panels, as well as a non-contrast CT of the brain and a CT of the abdomen and pelvis with contrast, which, aside from hepatomegaly with an elongated right hepatic lobe, were negative for any acute findings (Figure 1).

Given the patient's persistent fevers and myalgias, a decision was made to admit the patient to the hospital for blood culture follow-up and further workup. While in the hospital, the patient underwent an extensive evaluation, including consultations with an infectious disease specialist, blood cultures, an endoscopy, an MRI, a cholangiogram, and an MRI of the thoracolumbar spine, all of which were unremarkable. Lumbar puncture (LP) testing was considered, though given the absence of expected symptoms or signs for meningitis or encephalitis, it was felt that the diagnostic yield of an LP would be low, and it was decided rather to have a low threshold to pursue this if the patient were to have any persistent or worsening clinical findings. Additional laboratory testing was conducted on the first hospital day. Importantly, the patient was found to have positive DENV IgM and IgG assay values, which resulted prior to discharge on hospital day five. The Epstein-Barr virus (EBV) DNA PCR was also mildly elevated, along with a negative EBV-VCA (viral capsid antigen) IgM assay and a positive EBV-VCA IgG assay (Table 3).

**Table 3:** Relevant serum laboratory test values during hospitalization

Laboratory Test	Result	Reference Range
DENV IgM antibody	4.94 index	<1.65 index
DENV IgG antibody	1.8 index	<0.80 index
EBV-VCA IgM antibody	0.04 index	0.0–0.9 index
EBV-VCA IgG antibody	5.24 index	0.0–0.9 index
EBV DNA PCR	20 copies/PCR	<5 copies/PCR

His white blood cell and platelet counts reached nadirs of 2.41 and 66, respectively, over the course of a five-day hospitalization, all of which normalized by the time of hospital discharge. At this point, he experienced significant clinical improvement and remained afebrile for over 72 hours. The patient also did not develop any rash throughout his entire clinical course. The AST and ALT also peaked at 330 and 191, respectively, and subsequently returned to normal levels.

As noted earlier, the patient had frequent exposure to mosquitoes outside his home, which was thought to be the likely source of a suspected acute DENV infection, given the lack of recent travel to any endemic areas. Due to the concern for any possible murine typhus infection, he had been started on doxycycline and ceftriaxone empirically in the hospital and was de-escalated to doxycycline alone. This was done at the recommendation of the consulting infectious disease specialist, given their concern over the increased local risk for this. Upon discharge from the hospital, the patient was scheduled to follow up with a primary care physician and repeat laboratory testing in one to two weeks.

## Discussion

Our patient's presentation was most consistent with a locally acquired acute DENV infection due to a positive dengue IgM virus antibody, along with his overall clinical presentation, despite no recent travel to known endemic areas. In the acute setting, the evaluation of a patient with suspected DENV infection should concurrently rule out other potentially relevant high-risk febrile illness causes, such as bacterial sepsis, murine typhus, acute HIV infection, viral hepatitis, rickettsial infections, other viral hemorrhagic fevers, or EBV infection. This case report presents a patient with ongoing high fever, headaches, myalgias, and nausea. His test results were most notable for thrombocytopenia, lymphopenia, hepatomegaly, and abnormal liver enzymes. These are abnormalities that often occur in the setting of acute DENV infections [7]. While he also did not meet lab-based criteria for acute pancreatitis with his modest lipase elevation, there have been reports of pancreatitis as a rare complication of dengue fever [8]. The patient also noted no exposure to fleas that serve as typical vectors for *Rickettsia typhi*, which made murine typhus infection less likely. Furthermore, the CT scans did not demonstrate any splenomegaly or lymphadenopathy, which would be more expected with an acute EBV infection. Although the patient did have a mildly elevated EBV DNA PCR value, the IgM was negative, suggestive of a more remote EBV infection. The significant finding of a positive DENV IgM antibody aligns with a recent DENV infection, as defined by the National Notifiable Diseases Surveillance System (NNDSS), and our patient's clinical presentation, despite a lack of recent travel history to a country or region endemic for DENV [9].

During the acute phase of symptom onset, the CDC recommends a serum sample for dengue testing consisting of either a nucleic acid amplification test (NAAT) and an IgM antibody test or an NS1 enzyme-linked immunosorbent assay (ELISA) test and an IgM antibody test. According to the NNDSS, a positive result with NAAT or NS1 ELISA testing is a confirmatory laboratory criterion for dengue infection diagnosis [9]. Furthermore, patients with IgM antibodies against DENV are classified as having a presumptive, recent DENV infection. The antibody response of IgM can be detected within three to five days of illness onset and reaches peak levels approximately two weeks afterwards, making consideration of this lab test important for early detection and initiation of supportive therapeutic interventions [10].

Two similar cases of autochthonous DENV infection were reported by the Los Angeles County Department of Public Health, where two separate residents of Baldwin Park, California, were found to have locally acquired dengue infections with no history of travel to dengue-endemic areas prior to the onset of symptoms [11]. Notably, these incidents occurred near the same time as our case patient; however, the patient reported here did not travel to this locale, nor were there any other reports documented in his area. It is important to note that *Aedes *mosquitoes are common in Los Angeles County, but nearly all cases of diagnosed dengue in this area have been associated with travel to a country where dengue is prevalent [11,12]. There have also been previous reports of outbreaks in other non-endemic parts of the United States, such as Hawaii, of locally acquired cases of dengue fever, where the affected individuals and their family members had no travel history and reported nonspecific symptoms, including fever, headache, and myalgias [13]. These cases contribute valuable insights into the need for clinical vigilance in patients who present with febrile viral illnesses and mosquito exposure, despite no recent travel history to dengue-endemic areas.

## Conclusions

Our case is unique in that the patient's epidemiologic history did not follow the classic risk factors for DENV infections, which are primarily observed in individuals residing in or traveling to endemic areas. We highlight this case presentation for clinicians to consider possible DENV infection in the differential diagnosis of any patient who presents with an acute febrile illness, regardless of travel history. In addition, this case emphasizes the importance of vector control efforts for public health surveillance and maintenance. Given the availability of respiratory testing panels, PCR testing, and antigen testing, this additive testing should be considered in the evaluation of unexplained febrile illnesses.
